# Inferring Evolution of Habitat Usage and Body Size in Endangered, Seasonal Cynopoeciline Killifishes from the South American Atlantic Forest through an Integrative Approach (Cyprinodontiformes: Rivulidae)

**DOI:** 10.1371/journal.pone.0159315

**Published:** 2016-07-18

**Authors:** Wilson J. E. M. Costa

**Affiliations:** Laboratory of Systematics and Evolution of Teleost Fishes, Institute of Biology, Federal University of Rio de Janeiro, Rio de Janeiro, RJ, Brasil; Texas A&M University, UNITED STATES

## Abstract

Cynopoecilines comprise a diversified clade of small killifishes occurring in the Atlantic Forest, one of the most endangered biodiversity hotspots in the world. They are found in temporary pools of savannah-like and dense forest habitats, and most of them are highly threatened with extinction if not already extinct. The greatest gap in our knowledge of cynopoecilines stems from the absence of an integrative approach incorporating molecular phylogenetic data of species still found in their habitats with phylogenetic data taken from the rare and possibly extinct species without accessible molecular information. An integrative analysis combining 115 morphological characters with a multigene dataset of 2,108 bp comprising three nuclear loci (*GLYT1*, *ENC1*, *Rho*), provided a robust phylogeny of cynopoeciline killifishes, which was herein used to attain an accurate phylogenetic placement of nearly extinct species. The analysis indicates that the most recent common ancestor of the Cynopoecilini lived in open vegetation habitats of the Atlantic Forest of eastern Brazil and was a miniature species, reaching between 25 and 28 mm of standard length. The rare cases of cynopoecilines specialized in inhabiting pools within dense forests are interpreted as derived from four independent evolutionary events. Shifts in habitat usage and biogeographic patterns are tentatively associated to Cenozoic paleogeographic events, but the evolutionary history of cynopoecilines may be partially lost by a combination of poor past sampling and recent habitat decline. A sharp evolutionary shift directed to increased body size in a clade encompassing the genera *Campellolebias* and *Cynopoecilus* may be related to a parallel acquisition of an internally-fertilizing reproductive strategy, unique among aplocheiloid killifishes. This study reinforces the importance of adding morphological information to molecular databases as a tool to understand the biological complexity of organisms under intense pressure from loss of habitat.

## Introduction

The Atlantic Forest of eastern Brazil has been known since the nineteen century for its rich diversity of animal and plant species [1, 2, 3], and is placed among the five most important biodiversity hotspots in the world [4]. With considerable occurrence of endemic taxa, the Atlantic Forest is also one of the most endangered biodiversity hotspots [4, 5, 6]. Its biological richness has been correlated with its peculiar geographical position, consisting of a narrow stripe of forests along the tropical and subtropical Brazilian eastern coast, between the latitudes of about 4° and 32° S, and altitudes between the sea level and 2900 m [7]. The Atlantic Forest is highly heterogeneous in species composition and vegetation formations, containing moist evergreen forests, semi-deciduous dry forests, coastal open areas, and restinga forests in sandy soils [8, 9].

Among fish groups endemic to the Atlantic Forest, the aplocheiloid killifish tribe Cynopoecilini is remarkable for including among its members the smallest and most colourful fish species of the biome, as well as standing among the vertebrates most threatened with extinction in the Neotropical region [10, 11]. Most cynopoecilines are endemics of the Atlantic Forest, but a few species are found in the adjacent grass areas of southern Brazil and northeastern Uruguay [12]. Like some other South American and African aplocheiloid lineages, all the cynopoecilines uniquely live in temporary pools formed during the rainy seasons [10, 13], which occur between October and January and between March and May in the northern portion of their area of distribution, and between December and February and again between July and September in southern localities [10, 14]. During the dry seasons, fish die but eggs survive in embryonic diapause stage buried in the bottom substrate until the next rainy season [10]. Killifishes adopting this uncommon life cycle have been called annual killifishes, i.e., having a single generation by year [13]. Since field studies have recently demonstrated that two generations per year is the most common life cycle pattern, they could more appropriately be called ‘seasonal’ killifishes [14]. Cynopoecilines also include the only internally-fertilizing aplocheiloid killifishes [15, 16]. In addition, cynopoecilines are quite specialised in habitat use, with some species found in open vegetation savannah-like areas and others restricted to dense forest habitats, as described below.

The Cynopoecilini have been the focus of taxonomic studies [12, 17, 18, 19, 20, 21, 22] and some species have been included in phylogenetic analyses involving more inclusive groups using both morphological characters [15, 23] and mitochondrial DNA sequences [24, 25]. Morphological characters were not exhaustively sampled, since morphological analyses directed to cynopoeciline groups [20, 22, 26] were limited to characters described in previous papers, which focused on more inclusive groups [15, 23]. Moreover, a multi-gene phylogenetic analysis was restricted to taxa found in recent field studies [16].

Clear gaps in our present knowledge on cynopoecilines stem from the absence of an integrative approach reconciling molecular phylogenetic data of common extant species [16] with phylogenetic data of rare and possibly extinct species where molecular information is absent. Such an approach could provide valuable insight into understanding the evolution of this fish group. The objective of this study is to evaluate the evolution of habitat use and body size by using phylogenetic analyses of an enhanced set of morphological characters and DNA sequences.

### Diversity of cynopoeciline killifishes

The tribe Cynopoecilini comprises five genera and 21 nominal species [20, 21, 22]. The northernmost occurrence of the group corresponds to the recently established genus *Mucurilebias* Costa, 2014 [21], which includes a single species, *M*. *leitaoi* (Cruz & Peixoto, 1991), reaching only 22 mm of standard length (SL), endemic to northeastern Brazil (about 18° S) [21]. Its habitat is the temporary pools situated within the dense moist Tabuleiro forest of the Mucuri river basin [27], which consists of forested plains with deep and steep-sided river valleys. *Mucurilebias leitaoi* has not been found since 1988, after large portions of the original forest had been converted to cattle pastures [21, 28].

The genera *Leptolebias* Myers, 1952 and *Notholebias* Costa, 2008 are endemic to south-eastern Brazil coastal plains (between about 22°30’S and 25°30’S) and comprise small-sized species, most of which are threatened with extinction or possibly extinct [10, 21, 28, 29]. *Notholebias* comprises four similarly small species, rarely reaching 25 mm SL [17, 30], found in pools among coastal open restinga vegetation. These areas consist of savannah-like formations with wide areas mostly occupied by grasses and cattails, or in pools at the border of the adjacent semi-deciduous seasonal forest [17]. Two species of *Leptolebias*, *L*. *citrinipinnis* Costa, Lacerda & Tanizaki, 1988 and *L*. *opalescens* (Myers, 1942), are found in similar habitats as those described for *Notholebias* [31]. The remaining four species are uniquely found in shallow temporary channels and small pools within dense lowland moist forests at the base of the Serra do Mar mountain range [10, 20, 32, 33, 34]. Species of *Leptolebias* reach about 28 mm SL, with the exception of *L*. *marmoratus* (Ladiges, 1934), the type species of the genus, and *L*. *splendens* (Myers, 1942), both of which barely reach 23 mm SL [32, 34].

The two remaining genera, *Campellolebias* Vaz-Ferreira & Sierra, 1974 and *Cynopoecilus* Regan, 1912, differ from other cynopoecilines by occurring in southern subtropical areas and reaching a larger size (about 35–45 mm SL), as well as by containing internally fertilizing species, instead external as in all other aplocheiloid killifishes [15, 35, 36, 37, 38, 39]. After internal fertilization, females deposit eggs while swimming above the bottom [15]. The four similar species included in *Campellolebias* inhabit pools situated at the border of coastal forests, but sometimes they occur in open vegetation formations of the southern Brazilian coastal plains (between about 24°30’S and 28°30’S) [39]. *Cynopoecilus* is endemic to a vast area of southern Brazil and northeastern Uruguay (between about 28°30’S and 34°00’S), with most species found in open grassland formations, but sometimes in pools at the border of coastal forests (person. observ.). An exception is a species recently described, *C*. *notabilis* Ferrer, Wingert & Malabarba, 2014, living in shallow pools and channels within a dense semi-deciduous seasonal forest (person. observ.).

### Historical overview of cynopoeciline systematics

Monophyly of the group including *Campellolebias*, *Cynopoecilus*, and *Leptolebias* was first proposed by Costa [23], when *Leptolebias* was considered the sister group of a clade comprising the other two genera. Subsequently, the group was formally recognised as subtribe Cynopoecilina [40], but later considered full tribe Cynopoecilini [41, 42]. Costa’s [23] phylogenetic analysis had as focus the family Rivulidae and was based on morphological and behavioural characters. Characters analysed by Costa [23] were revised by Costa [15] in a new phylogenetic analysis of the Rivulidae, obtaining a similar result for cynopoeciline relationships. This same set of morphological characters was employed by Costa [20, 21, 26] to analyse relationships among species of *Campellolebias*, relationships of *Notholebias* with other cynopoecilines, and to infer the phylogenetic position of *Mucurilebias leitaoi*, respectively, and by Ferrer *et al*. [22] to analyse relationships of a new species of *Cynopoecilus*. Possibly as a result of using the same data set first proposed for a more inclusive group, all analyses provided similar results in supporting *Campellolebias* and *Cynopoecilus* as sister groups, and in more recent analyses, indicating *Notholebias* as the sister group of a clade containing all other cynopoecilines [20, 21, 22]. Molecular analyses using mitochondrial DNA were directed to more inclusive groups, sampling only a few terminal cynopoeciline taxa [24, 25], and a recent multigene phylogeny was restricted to extant taxa collected between 2013 and 2014 [16].

## Materials and Methods

### Ethics Statement

Methods used in this study were approved by the Ethics Committee for Animal Use of Federal University of Rio de Janeiro (CEUA-CCS-UFRJ, permit number: 01200.001568/2013-87). Euthanasia methods followed the guidelines of the Journal of the American Veterinary Medical Association (AVMA Guidelines) [43] and European Commission DGXI [44, 45] (see below).

### Taxon sampling

Terminal taxa were select to represent all the main cynopoeciline lineages. Among the 13 species selected, only nine (*Campellolebias brucei* Vaz-Ferreira & Sierra, 1974, *Cynopoecilus fulgens* Costa, 2002, *Cynopoecilus melanotaenia* (Regan, 1912), *C*. *notabilis*, *Cynopoecilus nigrovittatus* Costa, 2002, *Leptolebias aureoguttatus* (Cruz, 1974), *L*. *citrinipinnis*, *Notholebias fractifasciatus* (Costa, 1988) and *Notholebias minimus* (Myers, 1942)) have been currently collected in field studies, making possible to obtain tissues for molecular analyses. The remaining four species included in this study, *Campellolebias dorsimaculatus* Costa, Lacerda & Brasil, 1989, *L*. *marmoratus*, *L*. *splendens*, and *M*. *leitaoi*, were not found in recent field studies and are on the verge of extinction if not already extinct [11, 21]; these species were represented in the phylogenetic analyses by only morphological data. Out-group selection was directed to taxa representing different lineages of the three aplocheiloid families; out-groups included *Nematolebias whitei* (Myers, 1942), a basal member of the tribe Cynolebiini, the sister group of the Cynopoecilini [15, 23, 24, 25]; *Kryptolebias ocellatus* (Hensel, 1868), a basal representative of the Rivulidae [46]; *Nothobranchius guentheri* (Pfeffer, 1893), a nothobranchiid aplocheiloid; and *Aplocheilus lineatus* (Valenciennes, 1846), an aplocheilid aplocheiloid. Most material examined for morphological characters was available in the fish collection of Institute of Biology, Federal University of Rio de Janeiro (UFRJ). Specimens collected to complement morphological analysis and to obtain tissues for molecular studies were collected between 2012 and 2014, when all known localities from the whole area of distribution of the Cynopoeciline were sampled. Specimens were captured with small dip nets (40 X 30 cm). All specimens were euthanized just after collection in a buffered solution of ethyl-3-amino-benzoat-methansulfonat (MS-222) at a concentration of 250 mg/l, for a period of 10 minutes or more, until completely ceasing opercular movements. Specimens used in morphological studies were fixed in formalin just after collection, for a period of 10 days, and then transferred to 70% ethanol; specimens used in molecular analyses were fixed just after collection in absolute ethanol and later preserved in the same fixative. Collections were made with permits provided by ICMBio (Instituto Chico Mendes de Conservação da Biodiversidade). A list of material examined appears in S1 Appendix.

### Morphology

The morphological character analysis focused on internal characters (osteology of all parts of the body and myology of the anal-fin support), external characters (urogenital papilla, fins, jaws, frontal squamation, latero-sensory system, and contact organs), egg structure and colour patterns. Osteological characters were examined in specimens cleared and stained following Taylor & Van Dyke [47]. Terminology for osteological structures followed Costa [48], for muscles, Winterbottom [49], for frontal squamation, Hoedeman [50], and for cephalic neuromast series, Costa [51]. Data on egg morphology were taken from Costa [15], Fava & Toledo-Piza [52] and Costa & Leal [53]; terminology for chorion morphology followed Costa & Leal [53]. Data on colour patterns were obtained from direct examination of live individuals during collections, and photographs of both sides of live individuals, at least two males and one female per collection, taken in aquarium between five and 24 hours after collection. Due to the great overlapping of meristic and morphometric values exhibited by different cynopoeciline lineages, quantitative morphological characters were not included in the analysis. The list of morphological characters, following Sereno [54] for character statement formulation, is given in S2 Appendix, which also includes references for first character description, pertinent explanation on character variability, and when necessary, justification for exclusion of characters used in previous studies. Distribution of character states among terminal taxa appear in the data matrix of S1 Table. The abbreviation ch. used in the text followed by numbers are characters numbered as in S2 Appendix.

### DNA sequences

The phylogenetic analysis also included a set of partial sequences of three nuclear genes, comprising the glycosyltransferase 1 (*GLYT1*), ectodermal-neural cortex 1 (*ENC1*), and rhodopsin (*Rho*), which yielded strong phylogenetic signal at all tree levels of the cynopoeciline clade [16]. List of specimens and respective GenBank accession numbers appear in S2 Table. Methods used for DNA extraction, polymerase chain reaction (PCR), and sequencing are as described by Costa *et al*. [16].

### Phylogenetic analysis

The phylogenetic dataset containing morphological and molecular data was analysed using Maximum Parsimony (MP), which is the most commonly employed method when analysing morphological characters. The MP analysis was performed with TNT 1.1 [55]. It was primarily rooted on the aplocheilid taxon *Aplocheilus lineatus*, since the Aplocheilidae have been often considered to be the sister group of the clade comprising all other aplocheiloids [25, 46]; analyses were also alternatively rooted in *A*. *lineatus* and *N*. *guentheri* yielding the same topology. The search for most parsimonious trees was conducted using the ‘traditional’ search and setting random taxon-addition replicates to 10, tree bisection-reconnection branch swapping, multitrees in effect, collapsing branches of zero-length, characters equally weighted, and a maximum 1,000 trees saved in each replicate. Morphological character states were treated as unordered and autapomorphies were included in the data matrix in order to support diagnoses of monotypic genera. Molecular data were analysed giving equal weight to all sites. Sequences were aligned using Clustal W [56], after which the DNA sequences were translated into amino acids residues with MEGA 6.0 [57] to test for the absence of premature stop codons or indels. Branch support was assessed by bootstrap analysis, using a heuristic search with 1,000 replicates and the same settings used in the MP search. The morphological dataset was also analysed alone following the same methods.

The combined dataset was further submitted to a Bayesian Inference approach (BI), using Mr. Bayes 3.2 software [58]. For the morphological data partition, the model JC69 + G was used following Lewis [59]. Molecular data partitions and their respective evolutionary models followed Costa *et al*. [16]. The BI analysis was conducted with the following settings: two Markov chain Monte Carlo (MCMC) runs of two chains each for 1 million generations, a sampling frequency of 100. All parameters between partitions except topology and branch lengths were unlinked. The appropriate burn-in fraction and convergence of the MCMC chains were graphically assessed by evaluating the stationary phase of the chains using Tracer v. 1.5 [60]. The final consensus tree and Bayesian posterior probabilities (PP) were generated with the remaining tree samples after discarding the first 25% of samples as burn-in.

### Evolution of habitat usage and body size

Ancestral character state reconstructions under MP (maximum parsimony) and ML (maximum likelihood) were primarily performed to evaluate evolution of habitat usage and body size in cynopoecilines. Data on habitat were recorded during numerous collecting trips (over 200 expeditions) in 30 years of field studies (1984–2014). Two habitat kinds were considered in the analysis: pools inside dense forests and pools in open vegetation or at the border of forests (see Introduction above). The list of locations and respective habitats is presented in Table 1. Data on maximum body size were taken from revisionary studies [12, 17, 26], when all specimens of the Cynopoecilini available in museum collections were examined. TNT 1.1 was used for ancestral state reconstructions under MP, using algorithms for analysis of continuous characters [61] for body size, and Mesquite 3.02 [62], for reconstructions under ML, with both methods attaining the same results.

**Table 1:** List of locations and respective habitats of cynopoecilines.

Species	Location	Habitat
*Campellolebias brucei*	28°46’43”S 49°19’36”W	pool in open vegetation
	28°45’45”S 49°17’32”W	canal in open vegetation
	27°39’56”S 48°33’18”W	pool at forest border
*Campellolebias chrysolineatus*	26°23’00”S 48°40’00”W	pool at forest border
	26°24’46”S 48°38’23”W	pool at forest border
	26°24’33”S 48°38’31”W	pool at forest border
*Campellolebias dorsimaculatus*	24°40’00”S 47°26’04”W	pool at forest border
*Campellolebias intermedius*	about 24°20’S 47°35’W	pool among shrubs
*Cynopoecilus feltrini*	28°55’51”S 49°30’53”W	pool in open vegetation
	28°30’26”S 48°48’01”W	pool in open vegetation
*Cynopoecilus fulgens*	31°58’01”S 51°59’48”W	pool in open vegetation
	31°49’19”S 51°41’21”W	pool in open vegetation
	31°15’52”S 51°02’40”W	pool in open vegetation
	30°50’59”S 50°41’21”W	pool in open vegetation
	30°09’37”S 50°13’26”W	pool in open vegetation
	30°09’09”S 50°14’25”W	pool in open vegetation
	29°59’20”S 50°11’33”W	pool in open vegetation
	29°57’34”S 50°13’53”W	pool in open vegetation
*Cynopoecilus intimus*	29°56’33”S 53°42’24”W	swamp at gallery forest border
*Cynopoecilus melanotaenia*	32°04’33”S 52°15’54”W	pool in open vegetation
	32°04’15”S 52°15’51”W	pool in open vegetation
	32°07’40”S 52°11’03”W	pool among shrubs
	32°06’01”S 52°09’55”W	pool in open vegetation
	31°46’54”S 52°13’45”W	cattail swamp
	31°54’26”S 52°18’58”W	pool in open vegetation
	32°31’47”S 52°32’31”W	pool in open vegetation
	32°44’16”S 52°38’32”W	pool in open vegetation
	32°44’40”S 52°38’41”W	pool in open vegetation
	31°06’56”S 52°01’41”W	pool in open vegetation
	30°55’17”S 51°54’01”W	pool in open vegetation
	31°04’41”S 52°02’18”W	pool in open vegetation
*Cynopoecilus nigrovittatus*	30°03’27”S 51°46’33”W	pool at forest border
	29°56’20”S 51°46’00”W	pool in open vegetation
	29°49’21”S 51°21’09”W	pool in open vegetation
	29°40’13”S 51°25’32”W	pool in open vegetation
*Cynopoecilus notabilis*	30°05’48”S 50°51’06”W	channels within dense semi-deciduous forest
*Leptolebias aureoguttatus*	25°42’16”S 48°34’27”W	channels within dense rain forest
	25°40’42”S 48°30’13”W	channels within dense rain forest
	25°30’32”S 48°21’54”W	channels within dense rain forest
	25°04’25”S 47°55’27”W	channels within dense rain forest
	24°43’24”S 47°34’43”W	channels within dense rain forest
*Leptolebias citrinipinnis*	22°57’57”S 42°53’33”W	pool in open vegetation
	22°58’04”S 42°57’31”W	pool in open vegetation
*Leptolebias itanhaensis*	24°13’09”S 46°55’25”W	channels within dense rain forest
*Leptolebias marmoratus*	22°39’30”S 43°25’46”W	channels within dense rain forest
	22°38’09”S 43°15’57”W	channels within dense rain forest
*Leptolebias opalescens*	22°38’24”S 43°16’31”W	cattail swamp at border of forest
	22°39’55”S 43°26’24”W	cattail swamp
	22°51’06”S 43°26’16”W	pool among shrubs
*Leptolebias splendens*	22°38’09”S 43°15’57”W	channels within dense rain forest
	22°34’43”S 43°01’30”W	channels within dense rain forest
*Mucurilebias leitaoi*	18°06’05”S 39°39’47”W	pool within dense Tabuleiro forest
*Notholebias cruzi*	22°33’50”S 41°58’56”W	pool in open vegetation
	22°34’34”S 41°59’10”W	pool in open vegetation
*Notholebias fractifasciatus*	22°55’21”S 42°55’42”W	cattail swamp
	22°54’26”S 42°49’20”W	pool in open vegetation
	22°58’04”S 42°57’31”W	pool in open vegetation
*Notholebias minimus*	22°59’50”S 43°22’17”W	pool at Restinga forest border
	22°43’28”S 43°42’12”W	cattail swamp
	22°57’00”S 43°36’45”W	pool in open vegetation
	22°58’43”S 43°36’39”W	cattail swamp
*Notholebias vermiculatus*	22°51’53”S 42°33’15”W	pool in open vegetation
	22°51’19”S 42°34’10”W	pool in open vegetation
	22°56’16”S 42°40’23”W	pool in open vegetation

Evolution of habitat preference was further analysed with the Bayesian model for ancestral area reconstruction [63], implemented in RASP 3.02 [64], using the model F8l with gamma distribution and running the MCMC for 5 million generations, sampling every 1,000 trees, and discarding the first 500 trees (10%) as burn-in; considering that presently each species is restricted to a single habitat kind, the possible number of ancestral areas was restricted to one. A second analysis was performed to infer possible biogeographic events affecting the distribution of the cynopoecilines during their evolution, using the same methods and parameters. Four areas of endemism were used in this analysis, which have been delimited in previous studies involving different organisms occurring in the Brazilian Atlantic forest [9, 10, 18, 65]: A, the Tabuleiro forest and the adjacent coastal plains of northern Espírito Santo and coastal southern Bahia, eastern Brazil; B, the rain forest and adjacent coastal savanna of Rio de Janeiro, southeastern Brazil; C, the rain forest and adjacent coastal open formations of southern Brazil; D, the transitional zone between the Atlantic forest and the Pampas, in southern Brazil and northeastern Uruguay.

## Results

### Phylogeny

**Fig 1:**
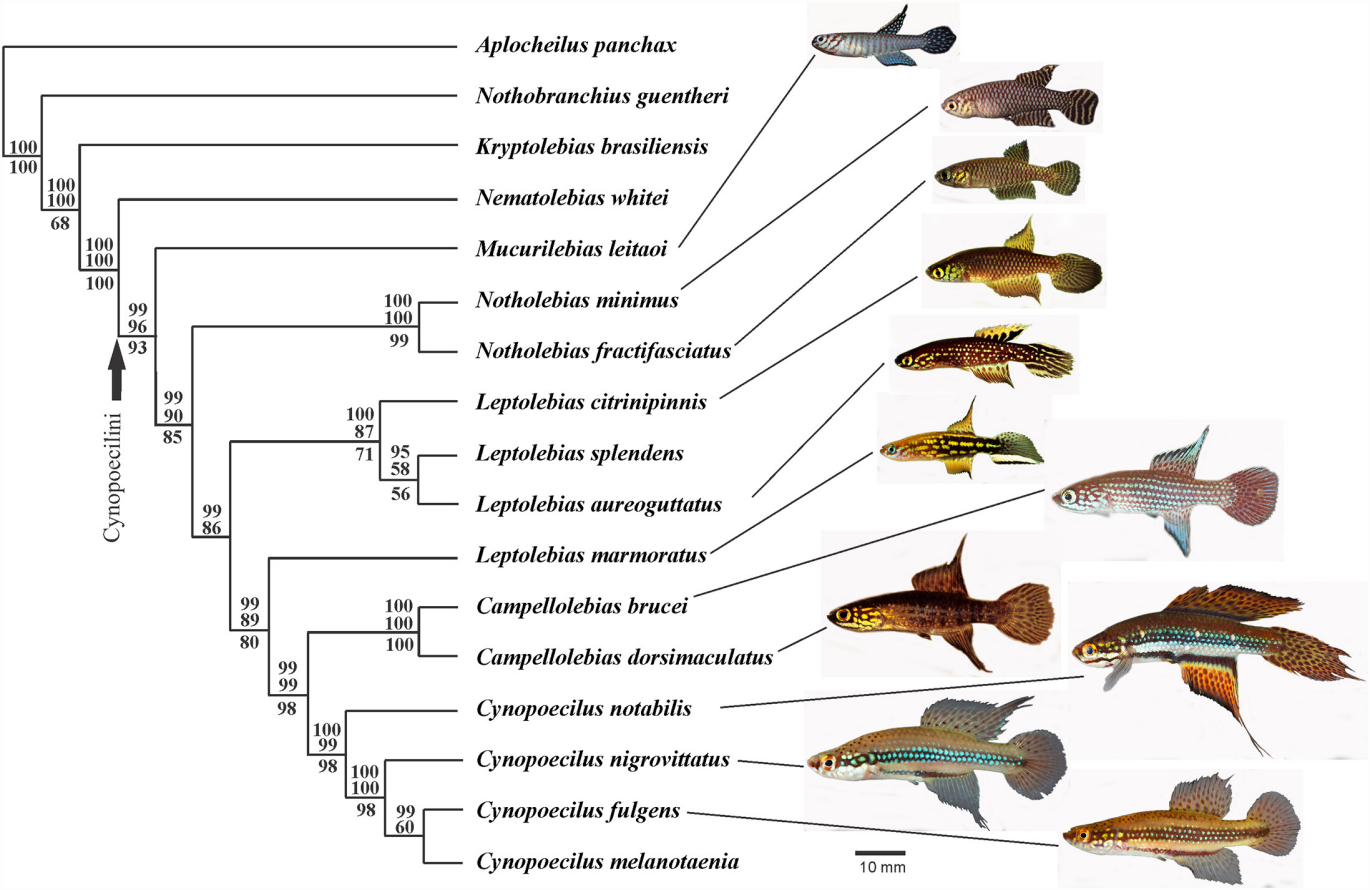
Phylogenetic relationships among 13 taxa of the Cynopoecilini and four out-group taxa. Single most parsimonious tree from the combined analysis of 115 morphological characters and a molecular data set, total of 2,108 bp, comprising segments of the nuclear genes *GLYT1*, *ENC1*, *Rho*. Numbers above branches are posterior probabilities of the Bayesian analysis higher than 95% (above) and bootstrap percentages of the maximum parsimony analysis higher than 50% (below), for the combined analysis; below branches, are bootstrap percentages higher than 50% for the analysis of morphological data alone. Photographs of male specimens were taken between five and 24 hours after field collection; unpaired fins are often damaged as a result of the strong aggressive behaviour occurring in cynopoeciline males.

The morphological data analysis generated 115 characters (see S2 Appendix for list of characters and S1 Table for distribution of character states among terminal taxa), of which 37 were new and 78 already described in previous studies [15, 18, 19, 20, 21, 23, 26, 46, 48, 66, 67, 68, 69]. Among the new characters, 16 were osteological, 15 referred to colour patterns, five to external morphology of fins and urogenital papilla, and one to cephalic latero-sensory system. The whole molecular dataset included 2,108 bp. The single most parsimonious tree generated in the analysis is depicted in Fig 1. An identical tree with lower bootstrap values was obtained when analysing morphological characters alone (Fig 1).

The resulting tree topology indicates relationships differing from that indicated in previous studies [15, 20, 21, 22], including *Mucurilebias leitaoi* as the sister group to a clade comprising all other cynopoecilines and *Leptolebias marmoratus* as the sister group to a clade comprising *Campellolebias* and *Cynopoecilus*.

### Evolution of habitat usage and body size

**Fig 2:**
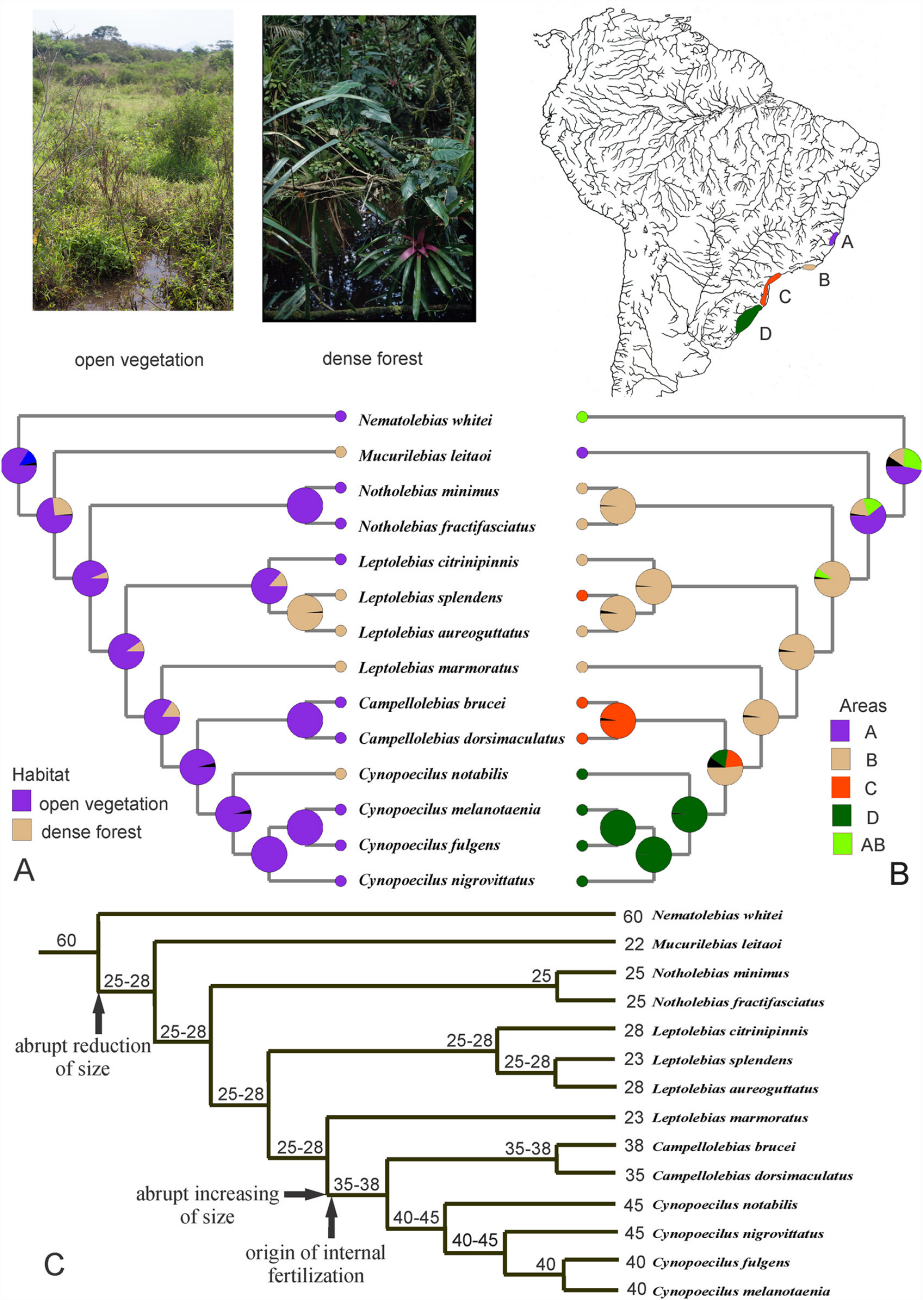
Evolution of habitat usage and body size in cynopoeciline killifishes. (A) Evolution of habitat usage, including open, savannah-like vegetation and dense forest, and (B) biogeographic reconstruction based on four areas of endemism for cynopoecilines, inferred from the Bayesian model for ancestral area reconstruction implemented in RASP 3.02, using the model F8l with gamma distribution, and maximum number of ancestral areas = 1 and 2, respectively; photographs above represent a typical costal open vegetation habitat and a dense rain forest habitat, and map illustrates geographic limits of areas of endemism. (C) Reconstruction of ancestral maximum standard length (values above branches), measured between snout and caudal-fin base (see also fish photographs in Fig 1 to see relative body size among different cynopoeciline lineages); numbers just before a species name refer to the maximum recorded standard length for that species.

All the performed analyses supported the same results (Fig 2). They indicated that the most recent common ancestor (MRCA) of the Cynopoecilini lived in open vegetation habitats (Fig 2A) of the coastal plains of the eastern Brazilian Atlantic forest (Fig 2B, area A) and was a miniature species, reaching between 25 and 28 mm SL (Fig 2C). Four independent evolutionary events were responsible for adaption to life in temporary pools within dense forests, whereas later an abrupt increase in body size occurred in a clade of internally-fertilizing species. The first event relative to the colonization of a dense forest habitat occurred in area A, which was followed by two other events in southeastern Brazil (area B), after dispersal of the ancestor of the clade comprising the genera *Notholebias*, *Leptolebias*, *Campellolebias* and *Cynopoecilus* from area A to B. Two lineages later independently dispersed to the southern Brazilian Atlantic forest (area C). One of these belonged to *Leptolebias*, living in dense forest, and another corresponded to the ancestor of the genera *Campellolebias* and *Cynopoecilus*, living in open vegetation areas. Finally, and more recently, the ancestor of *Cynopoecilus* dispersed further south, reaching the transitional zone between the Atlantic forest and the adjacent Pampas, when another lineage, corresponding to *C*. *notabilis*, adapted to the life within dense forest.

## Discussion

### Significance of an integrative approach to assess phylogenetic relationships of endangered organisms

Recent multi-gene phylogenetic analyses have consistently indicated new well-supported phylogenies of actynopterygian fishes, providing interesting new insights to understand relationships of historically problematic groups (*e*.*g*. [70, 71, 72, 73]). However, several taxa for which tissues are not available for DNA extraction have their phylogenetic position still unknown [16, 24, 25]. This situation is particularly problematic for taxa representing unique lineages that have gone extinct in recent decades, as occurring in cynopoeciline killifishes [11]. The recent well-supported multi-gene phylogeny of cynopoecilines [16] did not include two important taxa, *L*. *marmoratus* and *M*. *leitaoi*, which besides being type species of their respective genera, represent unique lineages among cynopoecilines [20, 21]. The results of the analysis presented herein, combining morphological data of three probably extinct miniature species with both morphological and molecular data of other extant species, although containing broad zones of missing information, generated a highly supported tree (Fig 1). This tree topology, in contrast to previous studies, supports *Leptolebias* as paraphyletic and *M*. *leitaoi* as the sister group of a clade comprising all other cynopoecilines.

### Habitat usage evolution

Cynopoecilines inhabits seasonal aquatic biotopes of the Atlantic Forest, a Neotropical biome that was shaped by climate changes during the Cenozoic (*e*.*g*. [74, 75]). According to the analysis herein, the MRCA of the Cynopoecilini lived in open vegetation habitats of the northern portion of the present distribution of the tribe (Fig 2A), as well as later lineage diversification was associated to long-range dispersal to south. Temporary pools in open vegetation areas constitute the most common habitat used by cynopoecilines today, including species of the genera *Notholebias*, *Leptopanchax*, *Campellolebias* and *Cynopoecilus* [31, 33, 39], despite dense forest being presently the predominant natural habitat in the region. These data highly suggest that at the time of cynopoeciline range expansion, open vegetation formations were more widespread than the present, making possible such long-range dispersal. Several studies have recorded both burst of species diversification [76, 77, 78] and range expansion of organisms living in open vegetation formations [79, 80] associated to paleogeographic episodes that occurred between the Oligocene and early Miocene of South America. At that time, the Andes was under an intense magmatic episode, making it the main relief in western South America and consequently acting as a rain shadow affecting South America climates, characterized by thermal optimum followed by a sharp glacial maximum [78]. In this cooler and dryer scenario, different organisms immigrated to new open habitats, expanding their geographic distribution [80]. However, the absence of fossil records for Neotropical aplocheiloids prevents a time-calibrated phylogeny for cynopoecilines to properly evaluate their timing of diversification.

The analysis also indicated that species in four cynopoeciline lineages became secondarily forest dwellers, uniquely living in temporary pools within the dense forest (Fig 2A). Forest dwellers are found in three disjunctive areas of dense forests, comprising the Tabuleiro forest of the Mucuri river basin, the rain forest of southeastern Brazil, and the semi-deciduous forest of southern Brazil (Table 1). Interestingly, these areas are separated by broad areas (about 600 km between the first and second areas, and about 550 km between the second and the third areas) where no forest dweller species are known, although substantial remnants of the original dense forest are still present. At first glance, this scenario is associated with the most recent drying episodes in the region that occurred during Pleistocene glaciations, when the dense forest retracted to form small isolated forested areas [81, 82], known as Quaternary forest refugia [83]. A recent study combining paleoclimatic modelling of forests with data on fossil pollen and phylogeography supports the presence of forest refugia in the Atlantic forest region at the Last Glacial Maximum, about 21,000 years ago [81]. The small and broadly separated areas inhabited by forest dwelling cynopoecilines may correspond to Quaternary forest refugia. However, the low frequency of forest dwellers among cynopoecilines may be the result of two other relevant factors, as discussed below.

The first is pertains to sampling. Forest dwelling cynopoecilines were rarely sampled historically, as a consequence of the difficult access of their habitat to fish collectors, i.e., shallow temporary channels in wet zones of the forest [34]. These habitats are typically concentrated in small areas of dense forest, making them difficult to find. *Leptolebias marmoratus* was not collected between 1944 and 2000, and *L*. *splendens*, between 1944 and 1985, even with frequent efforts by scientists and aquarists to find them [32, 34]. *Mucurilebias leitaoi* was collected only twice in 1988 [27], but it was not located during numerous subsequent collecting trips [21]. In addition, *C*. *notabilis* was first collected in 2011 [22], even though it is endemic to a region well explored by ichthyologists. The second relates to habitat destruction. The dense flooded forests of the coastal plains of eastern Brazil are among the habitats most severely extirpated in recent decades. These data suggest that forest-dwelling cynopoecilines were present in areas of dense forest that were not sampled for seasonal fish species in the past, but today have disappeared as a consequence of an intense deforestation.

### Miniaturization

Many miniature teleost fishes that barely reach about 25 mm of standard length as maximum adult size have been recorded for the Neotropical region [84, 85]. Among New World aplocheiloid killifishes, evolutionary events of miniaturization have independently occurred numerous times in different familial lineages [15]. However, the present study revealed that among cynopoecilines miniaturization was acquired a single time in the MRCA of the Cynopoecilini, and the condition was later reversed to a larger body (Fig 2C)

According to Weitzman & Vari [84], events of miniaturization in teleost fishes may result in errors in their phylogenetic placement, since reductive morphological characters often appear as homoplastic during the evolution of non-related miniature lineages. In agreement with such statement, *Leptolebias* was first diagnosed solely by the reduction of dark pigmentation in females [86], encompassing species presently placed in different genera of miniature species. However, in the present phylogenetic study, only eight of 115 characters involved reductive events within the Cynopoecilini clade (chs. 1.1; 3.1; 9.1–2; 16.2; 25.1; 34.1; 56.1; 77.1; 91.0), Consequently, the loss of phylogenetic signal is likely minimal.

The most striking shift in body size occurred in the MRCA of the clade comprising *Campellolebias* and *Cynopoecilus*. The change consists of a transformation from the cynopoeciline ancestral state of a small maximum size of about 25–28 mm SL to a considerable larger size, above 35 mm SL and gradually reaching to 45 mm SL (Fig 2C), the largest adult size among cynopoecilines. This shift coincides with the acquisition of internal fertilization in that clade (Fig 2C), indicating a possible relationship between insemination and body size increasing.

## Conclusion

This study consists of an integrative analysis combining an extensive dataset of morphological characters with a multigene dataset, which provided a robust phylogeny of cynopoeciline killifishes. This phylogeny is the first to support an accurate phylogenetic placement of nearly extinct species. This resulting well-corroborated phylogenetic tree supports a complex life history evolution, involving habitat specializations and miniaturization. This study thus reinforces the importance of adding morphological information to molecular databases as a tool to understand the biological complexity of life. More importantly, this integrative approach is fundamental in cases where organisms of interest involve recently extinct species, for which molecular data are unavailable.

## Supporting Information

S1 Appendix List of material examined for the analysis of morphological characters. (DOCX)

S2 Appendix List of character statements used to reconstruct the phylogenetic hypothesis amongst the Cynopoecilini. (DOCX)

S1 Table Data matrix of distribution of character states of 115 morphological characters among 17 terminal taxa. Character sequence follows S2 Appendix.
(DOCX)

S2 Table List of species used in the molecular analysis, and respective catalogue numbers and GenBank accession numbers. (DOCX)
