# Supplementary material for: Inferring Evolution of Habitat Usage and Body Size in Endangered, Seasonal Cynopoeciline Killifishes from the South American Atlantic Forest through an Integrative Approach (Cyprinodontiformes: Rivulidae)
Source: PLoS One. 2016 Jul 18;11(7):e0159315. doi: 10.1371/journal.pone.0159315 (PMC4948875; doi:10.1371/journal.pone.0159315)
Supplement: S1 Appendix — (DOCX) [file pone.0159315.s001.docx]

**S1 Appendix.** List of material examined for the analysis of morphological characters. C&S means specimens cleared and stained for osteological analysis, H, holotype, L, lectotype, N, neotype, P, paratype, S, syntype. Abbreviations for institutions are: BMNH, Natural History Museum, London; CAS (SU), California Academy of Sciences, San Francisco, formerly deposited in the Stanford University; MCP, Museu de Ciências e Tecnologia da Pontifícia Universidade Católica, Porto Alegre; MNRJ, Museu Nacional, Universidade Federal do Rio de Janeiro, Rio de Janeiro; MZUSP, Museu de Zoologia, Universidade de São Paulo, São Paulo; UFRJ, Instituto de Biologia, Universidade Federal do Rio de Janeiro, Rio de Janeiro; and, ZVC-P, Facultad de Humanidades y Ciencias, Departamento Zoología de Vertebrados, Montevideo.

**Genus *Campellolebias*:** *C. brucei*: Brazil: Estado de Santa Catarina: – ZVC-P 2126, 1 P; ZVC-P 2127, 1 P; near Criciúma, 28°46’43”S 49°19’36”W. – UFRJ 293, 7; UFRJ 1854, 4 (C&S); UFRJ 4493, 111; UFRJ 4494, 6 (C&S); near Criciúma, 28°45’45”S 49°17’32”W.– UFRJ 8382, 2; UFRJ 8383, 3; UFRJ 8374, 25; Florianópolis, 27°39’56”S 48°33’18”W. *C. chrysolineatus*: Brazil: Estado de Santa Catarina: – MZUSP 38817, H; MZUSP 38818, 2 P; MZUSP 38819, 1 P; MNRJ 11494, 2 P; MZUSP 38344, 11 P; near Araquari, 26°23’00”S 48°40’00”W. – UFRJ 5210, 6; UFRJ 5211, 2 (C&S); between Araquari and Balneário Barra do Sul, 26°24’46”S 48°38’23”W. – UFRJ 6317, 2; UFRJ 6318, 21; UFRJ 6319, 6 (C&S); UFRJ 10763; 2 (C&S); between Araquari and Balneário Barra do Sul, 26°24’33”S 48°38’31”W. *C. dorsimaculatus*: Brazil: Estado de São Paulo: – MZUSP 38813, H; MZUSP 38814, 1 P; MZUSP 38815, 12 P; MZUSP 38816, 1 P; MZUSP 11493, 2 P; MNRJ 11493, 2 P; UFRJ 6309, 2; UFRJ 6310, 3 (C&S); UFRJ 6311, 1; Iguape, 24°40’00”S 47°26’04”W. *C. intermedius*: Brazil: Estado de São Paulo: – UFRJ 6312, H; UFRJ 6313, 1 P; UFRJ 6314, 2 P; UFRJ 6315, 4 P (C&S); UFRJ 6316, 33; near Juquiá, about 24°20’S 47º35’W. **Genus** ***Cynopoecilus*:** *C. feltrini*: All from Estado de Santa Catarina, Brazil. – UFRJ 10662, H; UFRJ 10597, 22 P; UFRJ 10598, 6 P (C&S); UFRJ 10482, 5 P; UFRJ 10620, 7 P; Laguna, 28^o^30’26”S 48^o^48’01”W. – UFRJ 276, 6; UFRJ 1857, 2 (C&S); Araranguá, 28°55’51”S 49°30’53”W. – UFRJ 5234, 4; Sombrio. *C. fulgens*: All from Estado do Rio Grande do Sul, Brazil. – MCP 26929, H; MCP 26930, 5 P; UFRJ 5230, 5 P (C&S); 5 km N of São José do Norte, 31°58’01”S 51°59’48”W. – MCP 26933, H of *C. multipapillatus*; MCP 26934, 10; UFRJ 5232, 40; UFRJ 5233, 8 (C&S); UFRJ 4821, 118; near Lagoa da Fortaleza, 30°09’37”S 50°13’26”W. – UFRJ 10727, 54; UFRJ 10156, 6; near Osório, 29°57’34”S 50°13’53”W. – UFRJ 10174, 62; UFRJ 10157, 5; near Osório, 29°59’20”S 50°11’33”W. – UFRJ 10171, 62; UFRJ 10158, 4; near Cidreira, 30°09’09”S 50°14’25”W. – UFRJ 10704, 33; UFRJ 10159, 5; 35 km from Mostardas, 30°50’59”S 50°41’21”W. – UFRJ 10172, 84; UFRJ 10161, 4; Estreito, 31°49’19”S 51°41’21”W. – UFRJ 10726, 28; UFRJ 10160, 6; Tavares, 31°15’52”S 51°02’40”W. *C. intimus*: all from the upper Rio Jacuí drainage, Estado do Rio Grande do Sul, Brazil. UFRJ 4489, 13 P; UFRJ 4490, 6 P (C&S); Vila Block, 29°56’33”S 53°42’24”W. *C. melanotaenia*: Estado do Rio Grande do Sul, Brazil: BMNH 1909.9.5.15-22, 8 S; Estação Quinta. – UFRJ 4837, 14; UFRJ 5225, 4 (C&S); Estação Quinta, 32°04’33”S 52°15’54”W. – UFRJ 4980, 13; Estação Quinta, 32°04’15”S 52°15’51”W. – UFRJ 5033, 105 ex.; near Cassino, 32°07’40”S 52°11’03”W. – UFRJ 10703, 31; UFRJ 107402, 1 (C&S); UFRJ 10163, 7; between Quinta and Cassino, 32°06’01”S 52°09’55”W. – UFRJ 4008, 26; UFRJ 5228, 5 (C&S); Pontal da Barra, 31°46’54”S 52°13’45”W. – UFRJ 4979, 40; UFRJ 5019, 3 (C&S); near Pelotas, 31°54’26”S 52°18’58”W. – UFRJ 5058, 16; Taim, 32°31’47”S 52°32’31”W. – UFRJ 4947, 50; UFRJ 5223, 8 (C&S); near Taim, 32°44’16”S 52°38’32”W. – UFRJ 10729, 15; UFRJ 10162, 8; road BR-471, 32°44’40”S 52°38’41”W. – UFRJ 4498, 81; UFRJ 5224, 7 (C&S); near Cristal, 31°06’56”S 52°01’41”W. – UFRJ 4487, 32; UFRJ 4491, 4 (C&S); near Camaquã, 30°55’17”S 51°54’01”W. – UFRJ 10728, 3; UFRJ 10164, 5; near Cristal, 31°04’41”S 52°02’18”W. Uruguay: Departamento de Rocha: – UFRJ 10771, 10; Barra de Valizas, 34°19.81’S 53°48.89W. – UFRJ 10772, 14; Barra de Valizas, 34°19.34’S 53°49.36’W. – UFRJ 10773, 176; Arroyo Valizas, 34°21.55’S 53°50.64’W. – UFRJ 10774, 7; canal Andreoni, 33°55.21’S 53°32.61’W. – UFRJ 10776, 6; near Río Cebollatí, 33°36.82’S 54°18.00’W. – UFRJ 10775, 25; Lascano, 33°27.28’S 54°18.06W. Departamento de Trinta y Tres: – UFRJ 10769, 12; Paso de Dragón, 32°45.95’S 53°43.16W. – UFRJ 10770, 37; UFRJ 10778, 2; Paso de Dragón, 32°45.95’S 53°43.19’W. – UFRJ 10777, 40; near Arroyo Yerbal, 33°13.30’S 54°23.93’W. *C. nigrovittatus*: All from lower Rio Jacuí dranage, Estado do Rio Grande do Sul, Brazil. MCP 26931, H; MCP 26932, 10 P; UFRJ 4976, 45 P; UFRJ 5012, 6 P (C&S); Montenegro, 29^o^40’13”S 51^o^25’32”W. – UFRJ 4977, 23 P; UFRJ 5039, 46 P; Rio Caí floodplains, 29^o^49’21”S 51^o^21’09”W. – UFRJ 4820, 30 P; near São Jerônimo, 30^o^03’27”S 51^o^46’33”W. – UFRJ 4829, 43 P; UFRJ 5231, 6 P (C&S); between General Câmara and São Jerônimo, 29^o^56’20”S 51^o^46’00”W. *C. notabilis*: All from lower Rio Jacuí drainage, Estado do Rio Grande do Sul, Brazil. – UFRJ 10166, 4; UFRJ 10176, 4; UFRJ 10648, 2 (C&S); Banhado dos Pachecos, 30^o^05’48”S 50^o^51’06”W. **Genus *Leptolebias*:** *L. marmoratus*: Brazil: Estado do Rio de Janeiro: – MCP 28604, N; UFRJ 5404,1; UFRJ 5355, 24; UFRJ 5403, 1; UFRJ 5356, 4 (C&S); UFRJ 5220, 6 (C&S); Vila de Cava, 22°39’30”S 43°25’46”W. – MNRJ 4739, 8 S of *C. sicheleri*; CAS (SU) 36523, H of *C. zingiberinus*; CAS (SU) 36523, 10; road to Petrópolis, 22°38’09”S 43°15’57”W. *L. aureoguttatus*: Brazil: Estado do Paraná: – MNRJ 28924, L; MNRJ 19495, 2; UFRJ 199, 7; between Paranaguá and Matinhos, 25°42’16”S 48°34’27”W. – CAS (SU) 50191, 11; road east of Paranaguá. – UFRJ 6331, 11, UFRJ 6332, 5 (C&S); near Praia de Leste, 25°40’42” 48°30’13”W. Estado de São Paulo: UFRJ 5212, 35; UFRJ 5213, 10 (C&S); Iguape, 24°43’24”S 47°34’43”W. – MNRJ 19480, 7; MNRJ 19451, 8; MNRJ 19473, 6; MNRJ 19550, 2; Registro, about 24°31’S 47°51’W. *L. citrinipinnis*: Brazil, Rio de Janeiro: – MZUSP 37199, H; MNRJ 11310, 3 P; MZUSP 37198, 6 P; MZUSP 37197, 3 P; UFRJ 175, 1 (c&s); MNRJ 19432, 22; MNRJ 15400, 8;UFRJ 2202, 14; UFRJ 184, 2; UFRJ 2202, 14; UFRJ 3679, 3 C&S; UFRJ 3036, 5; UFRJ 4396, 10; UFRJ 4397, 5;UFRJ 4399, 6; UFRJ 4400, 13; UFRJ 4401, 2; UFRJ 4402, 7; UFRJ 10761, 3 (C&S); UFRJ 4403, 1; UFRJ 4404,11; UFRJ 4405, 5; UFRJ 5187, 19; UFRJ 6304, 16; UFRJ 6305, 4; UFRJ 8899, 4; UFRJ 8805, 10; UFRJ 8806, 15; Barra de Maricá, 22°57’57”S 42°53’33”W. – MNRJ 17684, 7; Itaipuaçu, 22°58’04”S 42°57’31”W. *L. itanhaensis*: Brazil, Estado de São Paulo: – UFRJ 6453, H; UFRJ 6323, 4 P; UFRJ 6324, 4 P; UFRJ 5219, 8 P (C&S); Itanhaém, 24°13’09”S 46°55’25”W. *L. opalescens*: Brazil: Rio de Janeiro: – CAS (SU) 36522, 6 P; MNRJ 11301, 3; road to Petrópolis, 22°38’24”S 43°16’31”W. – MNRJ 10621, H of *C. nanus*; MNRJ 10622, 1; MZUSP 25229/30, 2; Vila de Cava, 22°39’55”S 43°26’24”W. – UFRJ 9443, 6; UFRJ 9444, 10; Gericinó, 22°51’06”S 43°26’16”W. *L. splendens*: Brazil, Rio de Janeiro: – CAS (SU) 36528, 3 P; MNRJ 8514, 23; MNRJ 11302, 1; road to Petrópolis, 22°38’09”S 43°15’57”W. – MNRJ 11413, 3; MZUSP 38443, 2 (C&S); Citrolândia, 22°34’43”S 43°01’30”W. **Genus *Mucurilebias*:** *M. leitaoi*: Brazil: Estado da Bahia: – UFRJ 171, 1 (C&S); Mucuri, 18°06’05”S 39°39’47”W. **Genus *Notholebias*:** *N. cruzi*: Brazil: Estado do Rio de Janeiro: – MZUSP 36297, H; MZUSP 36298, 8 P; MNRJ 11289, 3 P; MNRJ 11290, 6 P; MNRJ 11291, 2 P; Barra de São João, 22°33’50”S 41°58’56”W. –UFRJ 173, 1 (C&S); UFRJ 5287, 3; UFRJ 2073, 6; Barra de São João, 22°34’34”S 41°59’10”W. *N. fractifasciatus*: Brazil: Estado do Rio de Janeiro: – MZUSP 36423, H; MZUSP 36424, 11 P; MNRJ 11292, 20 P; UFRJ 6452, 8 (C&S); Inoã, 22°55’21”S 42°55’42”W. – UFRJ 5358, 51; UFRJ 5357, 10 (C&S); Maricá, 22°54’26”S 42°49’20”W. *N. minimus*: Brazil: Estado do Rio de Janeiro. – UFRJ 4832, 44; UFRJ 6581, 10 (C&S); Bosque da Barra, 22°59’50”S 43°22’17”W. – MZUSP 36296, 11; MZUSP 36301, 3; MNRJ 11287, 4;UFRJ 6576, 10 (C&S); UFRJ 6575, 14; Seropédica, 22°43’28”S 43°42’12”W. – UFRJ 8269, 3; UFRJ 8268, 30; Campo Grande, 22°57’00”S 43°36’45"W. *N. vermiculatus*: Brazil: Estado do Rio de Janeiro: – UFRJ 8980, H; UFRJ 8898, 11 paratypes; UFRJ 8899, 3 P; UFRJ 8801, 6 P; near Sampaio Correia, 22°51’53”S 42°33’15”W. – UFRJ 8981, 1 P; UFRJ 8902, 10 P (C&S); UFRJ 8983, 8 P; UFRJ 8984, 60 P; near Sampaio Correia, 22°51’19”S 42°34’10”W. – UFRJ 8804, 6; UFRJ 8902, 4; UFRJ 8903, 23; UFRJ 8908, 4; near Jaconé, 22°56’16”S 42°40’23”W.
