# Supplementary material for: Inferring Evolution of Habitat Usage and Body Size in Endangered, Seasonal Cynopoeciline Killifishes from the South American Atlantic Forest through an Integrative Approach (Cyprinodontiformes: Rivulidae)
Source: PLoS One. 2016 Jul 18;11(7):e0159315. doi: 10.1371/journal.pone.0159315 (PMC4948875; doi:10.1371/journal.pone.0159315)
Supplement: S2 Appendix — (DOCX) [file pone.0159315.s002.docx]

**S2 Appendix.** List of character statements used to reconstruct the phylogenetic hypothesis amongst the Cynopoecilini.

**Osteology and myology**

**Superficial dermal bones and neurocranium**

[1] Dermosphenotic: (0) present; (1) absent (modified from Costa, 1990a: ch. 72).

[2] Vomer, posterior process, relative length to main portion of bone: (0) equal or slightly longer; (1) distinctively longer (modified from Costa, 1998: ch. 78). Remarks: Costa (1998: ch.78) considered the presence of an elongate posterior process of the vomer as a synapomorphy of *Campellolebias*, but present re-examination of this condition in large collections indicates that a relatively long and narrow process is present in all species of the genera *Campellolebias* and *Cynopoecilus*, including *‘C’. notabilis*. On the other hand, the apomorphic narrower lateral wings of the vomer previously assigned to occur in those two genera (Costa, 1998: ch.79) was not confirmed in the present study, since some variability was found in both genera, although the narrow wings being present in most specimens studied. Finally, an apomorphic narrow and long general shape of vomer represents a single evolutionary event as more precisely stated above when focusing on the posterior process.

[3] Vomer, teeth: (0) present; (1) absent (Costa, 1995a: ch. 8).

**Jaws, jaw suspensorium and opercular apparatus**

[4] Premaxilla and dentary, outer tooth row, larger teeth, arrangement: (0) along whole tooth patch; (1) concentrated on medial region (new character). Remarks: the apomorphic condition is only present in the genus *Notholebias*, in which the teeth are long and form a comb-like dentigerous structure in lateral view.

[5] Premaxilla, antero-proximal region, shape, and associated position of mouth cleft: (0) angular, mouth terminal; (1) slightly curved, mouth superior (modified from Costa, 2005: ch. 57, as in Costa, 2015: ch. 13).

[6] Maxilla, main axis, torsion: (0) not twisted; (1) slightly twisted (Parenti, 1981: unnumbered character).

[7] Maxilla, ventral process, distal expansion: (0) absent; (1) present (modified from Parenti, 1981).

[8] Dentary, coronoid process, extent relative to dorsal portion of angulo-articular: (0) extending above; (1) not extending above (Costa, 2011: ch.19).

[9] Angulo-articular, ventral process, shape: (0) well-developed, long; (1) short, well-visible in lateral view; (2) rudimentary, poorly or not visible in lateral view [not ordered] (modified from Costa, 1998: ch. 16).

[10] Autopalatine, ventral extremity, extent relative to dorsal portion of quadrate: (0) contacting; (1) not contacting (Costa, 1998: ch. 23).

[11] Autopalatine, median portion, constriction: (0) absent; (1) present (Costa, 1998: ch. 27). Remarks: Costa (1998) described a constriction on the median portion of the autopalatine as diagnostic for *Cynopoecilus*, but Ferrer *et al.*, 2014 found this character much variable, excluding it from the analysis. The apomorphic condition is here confirmed to occur in all examined species of all species of *Cynopoecilus*. This constriction is a consequence of a torsion at the middle portion of the bone, not always visible in lateral view, but conspicuous in posterior view.

[12] Autopalatine, general shape in lateral view: (0) nearly straight; (1) curved (new character). Remarks: representatives of the tribe and Cynolebiini typically have a nearly straight autopalatine, but in all cynopoecilines except *Mucurilebias* and *Cynopoecilus*, that bone is curved. Variable in *C. melanotaenia*.

[13] Autopalatine, postero-ventral process: (0) absent; (1) present (new character). Remarks: this process is present in all species of the Cynopoecilini, although rudimentary in *Cynopoecilus*.

[14] Autopalatine, postero-ventral process, length relative to antero-ventral process: (0) shorter; (1) longer (new character).

[15] Quadrate, posterior process, shape: (0) narrow; (1) wide (Costa, 1998: ch. 42). Remarks: Ferrer *et al.*, 2014 found this character much variable, excluding it from the analysis. The apomorphic condition is here confirmed to occur in all exemplars examined of all species of the Cynopoecilini. The apomorphic condition, however, is not always visible in a lateral view, being better observable in ventro-lateral view in the genera *Campellolebias* and *Cynopoecilus*.

[16] Mesopterygoid, extent relative to quadrate: (0) long, reaching metapterygoid and overlapping quadrate; (1) short, posterior tip in vertical through middle of quadrate, ventral portion slightly overlapping quadrate; (2) very short, posterior tip in vertical through anterior portion of quadrate (modified from Costa, 1990a: ch. 67 as in Costa, 2006a: ch. 5).

[17] Sympletic, shape: (0) short and deep; (1) long and narrow (modified from Costa, 1990a: ch. 49 as in Costa, 2006a: ch. 6). Remarks: the peculiar shape of the sympletic of the Cynopoecilini also involves a small dorsal expansion, making part of its dorsal margin in contact with the orbital margin.

[18] Preopercle, shape: (0) robust, L-shaped, with a well-developed anteromedian rim; (1) thin, C-shaped, with a reduced anteromedian rim (Costa, 1990a: ch. 4).

[19] Preopercle, dorsal portion, shape: (0) broad and rounded; (1) narrow and pointed (Costa, 1990a: ch. 56).

**Hyoid and branchial arches**

[20] Basihyal, shape: (0) broad, triangular; (1) narrow, about rectangular (Costa, 2008: ch. 10).

[21] Basihyal, length relative to length occupied by basibranchials: (0) shorter; (1) longer (new character).

[22] Urohyal, shape: (0) deep; (1) slender (Costa, 1995b: ch. 25).

[23] Interhyal, ossification: (0) ossified; (1) cartilaginous (Parenti, 1981: unnumbered character).

[24] Interarcual element, ossification: (0) cartilaginous; (1) ossified (new character).

[25] Second pharyngobranchial, teeth: (0) present; (1) absent (Costa, 2006a: ch. 10).

[26] Epibranchials, shape: (0) short; (1) long (Costa, 1998: ch. 60).

[27] Third epibranchial, uncinate process, shape: (0) narrow; (1) broad (new character).

[28] First hypobranchial, proximal edge, shape: (0) plain, terminating in single cartilage connected to second basibranchial; (1) bifid, terminating in cartilage connected to second basibranchial and another smaller cartilage connected to first basibranchial (Costa, 1998: ch. 70).

[29] First hypobranchial, distal edge, articular face extent: (0) restricted to cartilaginous head of first ceratobranchial; (1) anteriorly expanded (Costa, 2004: ch. 23.1).

[30] First hypobranchial, antero-lateral tip, anterior projection: (0) absent; (1) present (new character).

[31] Fourth ceratobranchial, teeth: (0) present; (1) absent (Parenti, 1981: unnumbered character).

[32] Fifth ceratobranchial, shape in dorsal view: (0) moderately robust, approximately triangular; (1) slender, boomerang-shaped (new character).

[33] Fifth ceratobranchial, anterior portion, tooth plate extent: (0) separated from extremity by space; (1) near anterior condyle (new character). Remarks: in the apomorphic condition occurring in *Notholebias*, the anterior portion of the fifth ceratobranchial is also slightly bent and bears a distinctive larger teeth, visible only in medial view, which is considered as a dependent condition.

[34] Fifth ceratobranchial, anterior portion, lateral flap: (0) present; (1) absent (new character).

**Vertebrae and caudal skeleton**

[35] Caudal vertebrae, neural pre-zygapophyses, development: (0) well-developed; (1) rudimentary (modified from Costa, 1998: ch. 100, as in Costa, 2006a: ch.16).

[36] Caudal vertebrae, last neural spine, postero-basal portion, small process bearing ligament attached to anterior tip of epural: (0) absent; (1) present (Costa, 1998: ch. 102). Remarks: most cynolebiasines and cynopoecilines have a small projection on the postero-basal portion of the last neural spine in front of the epural tip, but a formation of a distinctive process is only present in *Campellolebias*.

[37] Caudal skeleton, hypurals, fusion degree: (0) separate; (1) fused (modified from Costa, 1998: ch. 103).

[38] Caudal skeleton, epural and parhypural, proximal region: (0) broad and approximately straight; (1) narrow and curved anteriorly (Costa, 1998: ch. 105).

[39] Caudal skeleton, epural, distal portion, width relative to width of distal portion of parhypural: (0) approximately equal; (1) much wider (Costa, 2014: unnumbered character).

[40] Caudal skeleton, minute accessory caudal cartilages: (0) absent; (1) present (modified from Costa, 1998: ch. 107, as in Ferrer *et al.*, 2014: ch.27). Remarks: this minute cartilage is apparently present in all species of *Cynopoecilus*, although poorly or not stained with alcian blue in some specimens. This condition has not been recorded for any other aplocheiloid.

**Dorsal and anal-fin skeleton, and urogenital region**

[41] Dorsal fin, origin relative to anal-fin origin: (0) posterior; (1) anterior (Costa, 1990a: ch. 92). Remarks: uniquely among cynopoecilines, all species of *Campellolebias* and *Cynopoecilus* have the dorsal-fin origin anterior to the anal-fin origin as a consequence of an anterior placement of the former fin, as discussed in Costa (1998).

[42] Anal fin in males, anterior proximal radials, shape: (0) drop-shaped or sub-triangular; (1) rod-shaped (Costa, 1998: ch. 114).

[43] Anal fin in males, anterior proximal radials, proximal region, proximity: (0) separated by interspace; (1) in close proximity (new character). Remarks: in terminal taxa exhibiting the apomorphic condition, fin rays are closely positioned as a consequence of the close proximity of the proximal portion of the proximal radials.

[44] Anal fin in males, first three or four proximal radials, cartilaginous proximal region, shape: (0) short; (1) long (new character).

[45] Anal fin in males, anterior portion, series of seven to ten small unbranched rays connected to first two developed anal-fin proximal radials to form an inseminating fan: (0) absent; (1) present (modified from Costa, 1995b: ch. 21; Costa, 1998: ch. 131).

[46] Anal fin in males, anterior two rays, shape and relative position to posterior portion of anal fin: (0) thin, short, not isolated from the posterior part of the fin; (1) long, thickened, separated from the posterior part of the fin by a deep gap in the fin membrane just posterior to second ray to form an inseminating tube (modified from Costa, 1990a: ch. 99, 1998: ch. 156). Remarks: in all taxa exhibiting an inseminating tube, there are strong ligamentous connections between first two anal-fin rays and first three proximal radials, formerly considered as a separate character (Costa, 1998: 118), but here interpreted as a dependent condition.

[47] Anal fin in males, anterior two rays, basal portion, lateral sharp projections: (0) absent; (1) present (new character). Remarks: in taxa exhibiting the apomorphic condition, besides having the lateral projection on the basal portion, the two rays forming the inseminating tube are three or four times wider than adjacent posterior rays, as well as the first proximal radial has a pronounced lateral keel.

[48] Anal fin in males, sub-anterior portion, three to five branched rays connected to proximal radials 3-6, shape and relative position to posterior portion of anal fin: (0) straight, not separated from posterior portion of fin; (1) curved posteriorly, separated from posterior portion of fin by fin membrane gap (modified from Costa, 1995b: ch. 21; Costa, 1998: ch. 125).

[49] Anal fin in males, muscular support, anterior *inclinatores and depressores anales*, development: (0) tiny; (1) hypertrophied (modified from Costa, 1998: ch.186).

[50] Urogenital region in males, ejaculatory pump consisting of bulb-like structure formed by transverse muscular fibres: (0) absent; (1) present (Costa, 1998: ch. 185).

Excluded character: Anal fin in males, first proximal radial: (0) approximately straight; (1) curved posteriorly (Costa, 1995c). Remarks: first assigned for *Campellolebias*, the apomorphic condition is variable is this genus and in most other cynopoecilines, thus considered here as not informative.

**Shoulder and pelvic girdle**

[51] Pectoral fin, posttemporal, ventral process: (0) present; (1) absent (Costa, 1998: ch. 132).

[52] Pectoral fin, supra-cleithrum, shape: (0) short; (1) long (Costa, 1990a: ch. 73).

[53] Pectoral fin, cleithrum, posterior flange: (0) present; (1) absent (Costa, 1998: ch.135).

[54] Pectoral fin, radials, shape: (0) well-ossified, cub form; (1) thin, weakly ossified, disc shaped (Costa, 1990a: ch. 74).

[55] Pectoral fin, radials, relative extent between ventral pectoral-fin radial and coracoid: (0) wide; (1) narrow (Costa, 2006b: ch. 59).

[56] Pectoral fin, dorsal-most radial, development: (0) well-developed; (1) rudimentary or absent (new character).

[57] Pectoral fin, first post-cleithrum: (0) present; (1) absent (Parenti, 1981, unnumbered character).

[58] Pelvic fin, ischial process: (0) present; (1) absent (Costa, 2006b: ch. 61).

[59] Pelvic fin bones, relative medial position: (0) in contact; (1) separated (new character).

**External morphology**

**Urogenital papilla**

[60] Genital duct and opening in males, relative position to anal fin: (0) duct terminating anterior to anal fin, opening close to anal-fin origin; (1) duct extending along anterior margin of anal fin, opening near the tip of first anal-fin ray (modified from Costa, 1998: ch. 169).

[61] Genital opening in males, shape: (0) small orifice; (1) broad transverse aperture (modified from Costa, 1998: ch. 171).

[62] Urogenital papilla in males, attachment to anal fin: (0) free; (1) attached (Costa, 1998: ch. 170).

[63] Urogenital papilla in males, relative position to anal-fin origin: (0) separated by interspace; (1) in close proximity (new character).

[64] Urogenital papilla in males, basal portion, shape: (0) not distinctive; (1) forming pronounced wide structure (new character).

[65] Urogenital papilla in females: (0) minute gap; (1) prominent pocket-like structure overlapping anterior anal-fin origin (Costa, 1998: ch. 172).

**Fins**

[66] Dorsal and anal fins in males, posterior extremity, shape: (0) rounded; (1) pointed (modified from Costa, 2006b: ch.68–69).

[67] Dorsal fin in males, tip, fin ray extension: (0) absent; (1) present (modified from Costa, 2006b: ch. 71).

[68] Anal fin, basal portion, thickened tissue (possibly glandular): (0) absent; (1) present (Costa, 1995b: ch. 19).

[69] Anal fin in males, general shape: (0) about rectangular, anterior and distal margins of fin well-delineated; (1) about triangular, anterior and distal margins nearly continuous (modified from Costa, 2006a: ch.36).

[70] Anal fin in males, tip, long filamentous ray: (0) absent; (1) present (new character).

[71] Pelvic fin in males, posterior extent: (0) anal fin; (1) urogenital papilla (modified from Costa, 1995b: ch. 20).

[72] Pelvic fin in males, shape: (0) nearly drop-shaped; (1) sub-triangular (new character).

[73] Caudal fin in males, shape: (0) oval, slightly longer than deep; (1) round, about so long as deep; (2) subtruncate; (3) lanceolate; (4) asymmetrical, dorsal margin slightly rounded, ventral margin straight (new character). Remarks: Costa (2008) considered a caudal fin much longer than deep as a synapomorphy of *Leptolebias*. However, when comparing the several morphological patterns of caudal fin morphology in aplocheiloid lineages, it is difficult to assume that the oval caudal fin of *L. aureoguttatus* or *L. citrinipinnis* is homologous to the long asymmetrical caudal fin of *L. marmoratus*. This new character statement recognises five different but well delimited character states.

Excluded characters: Anal-fin membrane in male (Costa, 1998): (0) continuous; (1) interrupted to isolate anterior portion of fin. Remarks: the membrane gap occurring in species of *Campellolebias* and *Cynopoecilus* are situated in different position, thus considered non-homologous in the two genera. The gap occurring in *Campellolebias* is dependent of the presence of a mobile inseminating tube (character 39 above) and the other one in *Cynopoecilus* is dependent of the presence of a inseminating flap (character 41 above). – Pelvic-fin bases, relative medial position: (0) in close proximity; (1) separated by short interspace (Costa, 2006a: ch. 37). Remarks: this character is very variable among cynopoecilines, but accurately delimited when focusing on the relative position of the pelvic bone (character 51 above).

**Jaws and branchiostegal region**

[74] Opercular and branchiostegal membrane, relative position: (0) completely separated; (1) partially separated, gap posteriorly ending near middle of preopercle (Costa, 2005: ch.60).

[75] Jaws, shape and ventral branchiostegal gap shape: (0) long and broad, gaps between opercular and branchiostegal membranes parallel; (1) short and narrow, gaps between opercular and branchiostegal diverging anteriorly (Costa, 2015: ch. 8).

**Frontal squamation**

[76] E-scales, relative medial position: (0) overlapped; (1) not overlapped (Costa, 1998: ch. 175).

[77] Supraorbital squamation, development: (0) well developed; (1) rudimentary or absent (Costa, 1998: ch. 176). Remarks: In previous studies (Costa, 1998, 2008), the Cynopoecilini were diagnosed by a rudimentary supraorbital squamation, a condition herein confirmed. However, Costa (2006a) equivocally diagnosed the Cynopoecilini by the absence of supraorbital scales, what was followed by Ferrer *et al.* (2014). In fact, a rudimentary supraorbital squamation comprising one minute scales is present in several cynopoecilines, but scales are eventually inconspicuous by being covered by dense epidermis, or even absent in small specimens.

**Neuromasts**

[78] Supraorbital series, anterior and posterior sections, arrangement: (0) separate; (1) continuous (Costa, 1998: ch. 180).

[79] Supraorbital series, interruption at level of posterior nostril: (0) absent; (1) present (new character). Remarks: this character is only applicable to taxa with continuous supraorbital series, in which it is possible to note a gap in the distribution of neuromasts. In *Cynopoecilus* and *Campellolebias*, the broad gap occurring in other cynopoecilines is absent, although a slight gap may be present in most specimens.

[80] Supraorbital series, neuromasts anterior to interrupted zone: (0) 2; (1) 1 (modified from Costa, 2008: ch. 52). Remarks: Costa (2008) considered the number of neuromasts in the anterior part of the supraorbital series without considering the distribution of neuromasts in the whole series. This character is thus only applicable to taxa exhibiting a gap in the supraorbital series close to the posterior nostril.

[81] Caudal fin, base, neuromasts, number: (0) 1–2; (1) 4–7 (Costa, 1998: ch.184).

**Contact organs**

[82] Flank, scale margin: (0) absent; (1) present (Costa, 2006b: ch. 87).

[83] Pectoral fin, inner surface: (0) absent; (1) present (Costa, 2006b: ch. 88).

**Egg morphology**

[84] Chorion, surface: (0) plain to verrucate; (1) reticulate (modified from Costa, 1990a: ch. 85).

[85] Chorion, surface, mushroom-like chorion projections: (0) absent; (1) present (modified from Costa, 1990a: ch. 85).

**Colour patterns**

[86] Flank in males, iridescent colour, arrangement pattern: (0) over whole flank; (1) restricted to midline of body; (?) taxa without iridescent marks (new character).

[87] Flank in males, iridescent marks, longitudinal arrangement: (0) continuous rows along flank; (1) fragmented rows along flank; (2) isolated in alternated scales to form vertical zigzag bars (modified from Costa, 2008: ch. 58).

[88] Flank in males, iridescent marks, colour: (0) light blue to yellowish green; (1) canary yellow (new character)

[89] Flank in males, lateral midline, broad dark reddish chocolate brown to black stripe: (0) absent; (1) present (modified from Costa, 1990a: ch. 95, as in Costa, 1998: ch. 190). Remarks: Costa (1990a: ch. 95) first used the presence of three longitudinal dark stripes on the flank as an apomorphic condition, diagnostic for *Cynopoecilus*. This character was then based only on the examination of preserved specimens and refers to three longitudinal zones of melanophores, comprising a broad mid-lateral stripe, a narrow stripe between the pectoral-fin base and the posterior portion of the anal-fin base, and a narrow row of melanophores on the middle of the dorsal portion of the flank. The presence of a broad mid-lateral longitudinal black stripe was considered in a distinct character in Costa (1998), what is followed here. The apomorphic condition is clearly seen in all species of *Cynopoecilus*, but not in other taxa. However, the entire flank of *L. marmoratus* exhibits the same colour (dark reddish chocolate brown), making the condition ambiguous in this taxon.

[90] Flank in males, ventral portion between pectoral-fin base and posterior portion of anal-fin base, dark reddish chocolate brown to black stripe: (0) absent; (1) present (modified from Costa, 1990a: ch. 95, as in Costa, 1998: ch. 191). Remarks: this stripe was considered diagnostic for *Cynopoecilus*, but besides occurring in all species of *Cynopoecilus* it is also present in *L. marmoratus*. However, although not visible in live specimens of species of *Campellolebias*, a similar stripe with the same position is conspicuously visible in recently preserved specimens.

[91] Flank in females, dark pigmentation: (0) rudimentary or absent; (1) present, forming distinctive dark marks (modified from Costa, 1990a: ch. 87).

[92] Flank in females, dark bars: (0) absent; (1) present (modified from Costa, 2006b: ch. 111).

[93] Dorsum in males, anterior portion, brown spots: (0) absent; (1) present (new character).

[94] Venter in males, middle zone, longitudinal dark brown stripe: (0) absent; (1) present (Costa, 2006a: ch. 50).

[95] Caudal peduncle in females, posterior-most portion, middle zone, black spots: (0) absent; (1) present (Costa, 2006a: ch. 52).

[96] Head in males, latero-ventral portion, stripe: (0) absent; (1) present (Costa, 2006a: ch. 51).

[97] Head in males, opercular region, three dark red bars (orbital, pre-opercular, opercular): (0) absent; (1) present (new character).

[98] Head in males, side, ascending oblique red stripe connecting orbital and opercular bars at level of ventral margin of orbit: (0) absent; (1) present (new character).

[99] Head in males, side, descending oblique dark reddish brown stripe connecting orbital bar and pectoral-fin base: (0) absent; (1) present (new character).

[100] Head in males, side, dark reddish brown pigmentation extending between orbit and posterior limit of head: (0) absent; (1) present (new character).

[101] Head in males, side, dark reddish brown pigmentation between lower jaw and orbit: (0) absent; (1) present (new character).

[102] Head in males, branchiostegal region, intense red pigmentation: (0) absent; (1) present (Costa, 2014: unnumbered character).

[103] Iris, bar through centre of eye: (0) absent; (1) present (Parenti, 1981, unnumbered character).

[104] Iris in males, colour: (0) yellow to yellowish brown; (1) green; (2) blue (modified from Costa, 2006a: ch.48, as in Costa, 2008: ch.62).

[105] Unpaired fins in males, distinctive dark red marks: (0) absent; (1) present (new character)

[106] Unpaired fins in males, distinctive white dots: (0) absent; (1) present (new character).

[107] Dorsal fin in males, distal zone, narrow bluish white stripe: (0) absent; (1) present (new character).

[108] Dorsal fin in males, distal zone, broad iridescent golden stripe: (0) absent; (1) present (new character).

[109] Dorsal fin in males, basal portion, brownish red stripe: (0) absent; (1) present (new character).

[110] Dorsal and anal fins in males, sub-distal region, distinctive concentration of melanophores: (0) absent; (1) present (Costa, 2006a: ch. 49).

[111] Dorsal fin in males, extent of dark red marks: (0) reaching antero-distal margin; (1) not surpassing sub-distal portion; (?) no dark red marks (modified from Costa, 2008: ch. 64).

[112] Dorsal fin in males, dark red pigmentation pattern: (0) small round spots; (1) anteriorly inclined bars (new character); (?) no dark red marks.

[113] Anal fin in males, distal margin, distinctive narrow red stripe: (0) absent; (1) present; (?) taxa with predominantly red anal fin (Costa, 2014: unnumbered character).

[114] Caudal fin in males, dark red pigmentation pattern: (0) small round spots on most portion of fin, often lines parallel to fin rays on posterior portion of fin; (1) narrow bars; (2) vermiculate marks; (?) no dark red marks (new character). Remarks: A similar character state distribution among taxa is present when analysing the dark red pigmentation of the anal fin, thus considered as a dependent character.

[115] Caudal fin in males, ventral portion, broad white stripe ventrally bordered by narrow black stripe: (0) absent; (1) present (new character).

Excluded characters:

Head in males, opercular region, red pigmentation pattern: (0) not patterned; (1) bars; (2) stripes; (3) reticulate (Costa, 2008: ch. 68). – Caudal fin in males, iridescent colour pattern: (0) dots; (1) bars or vertical lines; (2) vermiculate marks; (3) iridescence homogeneously arranged over fin; (4) horizontal lines; (?) no iridescence (Costa, 2008: ch. 65). Remarks: These two characters are here considered to involve different characters among their character states (*i.e.*, independent transformations not resulting in comparable homologous conditions), each being then divided in separate characters (see above).
