# Supplementary material for: Inferring Evolution of Habitat Usage and Body Size in Endangered, Seasonal Cynopoeciline Killifishes from the South American Atlantic Forest through an Integrative Approach (Cyprinodontiformes: Rivulidae)
Source: PLoS One. 2016 Jul 18;11(7):e0159315. doi: 10.1371/journal.pone.0159315 (PMC4948875; doi:10.1371/journal.pone.0159315)
Supplement: S1 Table — Character sequence follows S2 Appendix. (DOCX) [file pone.0159315.s003.docx]

**S1 Table.** Data matrix of distribution of character states of 115 morphological characters among 17 terminal taxa. Character sequence follows S2 Appendix.

*Aplocheilus panchax*

0000000000000?0000000100000000000000000000000000000000000000000000000000000000??000000000000000000000000000000??0?0

*Nothobranchius guentheri*

0000100100000?00000000000100000000001000100000000010?0000000000000000000211010??010000000000000000000002000000??0?0

*Kryptolebias ocellatus*

0000111101000?0001000000000100000000000000000000000000001000000010000000011000??000000?00010000000000000000000??0?0

*Nematolebias_whitei*

0000111101000?01011000101100111000101100000000000011111011000000111000001110010?001000?00011001010000010010000??0?0

*Mucurilebias_leitaoi*

1010111121001012111000101100111000101110000000000011111011000000111000101111110?011??0000000000010000110011000??1?0

*Notholebias_fractifasciatus*

0011111121011112111110100100101011101100000000000011111011000000111000001111111011111000000000001000001110000001010

*Notholebias minimus*

0011111121011112111110100100101011101100000000000011111111000000111000001111111011111000000000001000001110000001010

*Leptolebias citrinipinnis*

1010111121011012111010101100101001101100000000000011111011000000111000000111111101011000000000001000001110010011020

*Leptolebias_aureoguttatus*

1010111121011012111010101100101001101100000000000011111011000000111000000111111111011010000000001100001110010011020

*Leptolebias_splendens*

1010111121011012111010101100101001101100000000000011111011000000111000000111111101011020000000001100001210010011?20

*Leptolebias_marmoratus*

1010111121011012111010101100101001101100001000000011111011000011111100104111111101011011?10000001000001210001010001

*Campellolebias_dorsimaculatus*

1110111111011012111010101100101101111100111001101111111011110111111010012111110?110100?0011001111011101110000110000

*Campellolebias_brucei*

1110111111011012111010101100101101111100111001001111111011110111111010012111110?11010000011001111011101110000110000

*Cynopoecilus notabilis*

1110111111101012111110110110101001101101111010001111111011101011111101113111110?11011100111100001011011010001000000

*Cynopoecilus melanotaenia*

11101111111?1012111010100100101001101101111110011111111011101011111100111111010?11011100111010001001101010000010000

*Cynopoecilus_nigrovittatus*

1110111111101012111011100100101001101101111110011111111011101011111100111111010?11011100111010001001101010000010000

*Cynopoecilus_fulgens*

1110111111101012111010100100101001101101111110011111111011101011111100111111010?11011100111010001001101010000010000
