# Supplementary material for: Inferring Evolution of Habitat Usage and Body Size in Endangered, Seasonal Cynopoeciline Killifishes from the South American Atlantic Forest through an Integrative Approach (Cyprinodontiformes: Rivulidae)
Source: PLoS One. 2016 Jul 18;11(7):e0159315. doi: 10.1371/journal.pone.0159315 (PMC4948875; doi:10.1371/journal.pone.0159315)
Supplement: S2 Table — (DOCX) [file pone.0159315.s004.docx]

**S2 Table.** List of species used in the molecular analysis, and respective catalogue numbers and GenBank accession numbers.

Species Catalog number GenBank (ENC1; Glyt; Rho)

*Aplocheilus lineatus --------------* KC701974; KJ696985;

KC702042

*Nothobranchius guentheri* UFRJ 8418 ------------; KJ179338;

KT590088

*Kryptolebias ocellatus* UFRJ 9294 KC702011; KT590077;

KC702076

*Nematolebias whitei* UFRJ 6844 KC701991; KT590078;

KC702057

*Notholebias minimus* UFRJ 8841 KC701998; KT590079;

KT590089

*Notholebias fractifasciatus* UFRJ 8802 KT590070; KT590080;

KT590090

*Leptolebias citrinipinnis* UFRJ 8805 KT590071; KT590081;

KT590091

*Leptolebias aureoguttatus* UFRJ 10638 KT590072; KT590082;

KT590092

*Campellolebias brucei* UFRJ 8383 KC701980; KT590083;

KC702047

*Cynopoecilus* *melanotaenia* UFRJ 8974 KT590076; KT590087;

KT590096

*Cynopoecilus* *nigrovittatus* UFRJ 10165 KT590074; KT590085;

KT590094

*Cynopoecilus* *notabilis* UFRJ 10166 KT590075; KT590086;

KT590095

*Cynopoecilus* *fulgens* UFRJ 10160 KT590073; KT590084;

KT590093
